# Enhancing Set‐Type Yoghurt With Olive Leaf Extract: A Comparison of Powdered Addition and Surface Spraying on Quality, Functionality, and Sensory Attributes

**DOI:** 10.1002/fsn3.70273

**Published:** 2025-05-13

**Authors:** Büşra Deniz Aydın, Çiğdem Konak Göktepe, Nihat Akın

**Affiliations:** ^1^ Department of Food Engineering, Faculty of Agricultural Selcuk University Konya Türkiye; ^2^ Department of Food Processing, Karapınar Aydoğanlar Vocational School Selcuk University Konya Türkiye

**Keywords:** antioxidant activity, microbiological quality, olive leaf extract, sensory properties, yoghurt

## Abstract

In this study, the effects of using powdered olive ( *Olea europaea* L.) leaf extract (POLE) in yoghurt at different treatments and concentrations on the physicochemical, textural, functional, microbiological, and sensorial aspects of yoghurt were investigated. Accordingly, the enrichment of yoghurts with POLE was carried out by spraying aqueous solutions of POLE (0.4% and 0.6% solution) on the yoghurt surface after fermentation and adding POLE (0.4% and 0.6% w/v) to yoghurt before fermentation. While both POLE treatments caused no difference in the total solids, fat, protein, and ash contents of yoghurt, yoghurts sprayed with olive leaf extract aqueous solution exhibited similar results to the control regarding color and textural properties. Nevertheless, yoghurt enriched with POLE before fermentation showed higher total phenolic content and antioxidant properties. POLE treatments of yoghurt provided the expected antimicrobial effect without causing any change in the viable counts of yoghurt bacteria among the samples during 28 days of storage compared to plain yoghurt. Furthermore, surface spraying of POLE aqueous solution prevented the occurrence of an undesirable bitter taste in yoghurt resulting from the addition of POLE before fermentation. These results reveal that olive leaf, which is a cost‐free waste product of the olive industry, can be used as a potential antimicrobial and functional agent. In addition, the positive sensory results obtained by spraying POLE aqueous solution on the surface after fermentation suggest that this application may be an alternative method for using herbal extracts.

## Graphical abstract

The effects of powdered olive leaf extract (POLE) on yoghurt were examined. Yoghurt enriched with POLE before fermentation showed higher antioxidant properties. POLE treatments of yoghurt provided an antimicrobial effect during storage. Surface spraying of POLE aqueous solution after fermentation prevented bitter taste.

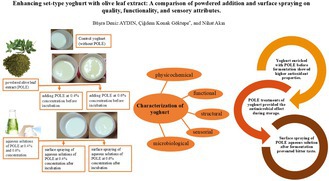

## 1 Introduction

Yoghurt is a widely consumed universal fermented dairy product that is produced by two bacteria named *Streptococcus* (*S*.) *thermophilus* and *Lactobacillus* (*L*.) *delbrueckii* subsp. *bulgaricus*. It plays an important role in human nutrition and health benefits with its valuable proteins, vitamins, minerals, and lactic acid bacteria (Gómez‐Gallego et al. 2018). In recent years, the supplementation of yoghurt with plant extracts has attracted great interest. As is known, traditional yoghurt is deficient in many bioactive components such as phenolics, vitamin C, and fibers (Lisak Jakopović et al. 2022). In addition, the rising demand for natural compounds instead of synthetic chemical molecules is also very effective in increasing the use of plant materials in yoghurt (Granato et al. 2018). In this context, milk‐based formulations containing different herbal extracts have been developed by many researchers to improve the functional properties of yoghurt (Mohamed Ahmed et al. 2021; Pourghorban et al. 2022; Zhang et al. 2019).

Olive leaves are an agricultural and industrial by‐product of the pruning of the olive tree (accounting for about 25% of its total weight) or the cleaning and sorting of the olive fruit before processing into oil (accounting for about 10% of the total weight of olives) (Markhali et al. 2020). A broad field of research is emerging, focusing on food applications, pharmaceuticals, and cosmetics to recover bioactive compounds from these by‐products, and on zero waste awareness. Phenolic compounds obtained from olive leaves are frequently used in biodegradable films and functional food applications in food packaging due to their antimicrobial and antioxidant properties (Nunes et al. 2016). The phytochemical profile of olive leaves draws attention to their potential for use in the food industry as it improves the shelf life of foods and enhances food quality and their positive effects on health (Difonzo et al. 2021; Kermanshah et al. 2020; Nasrollahi and Abolhasannezhad 2015). Furthermore, many studies show that olive leaves, rich in phenolic compounds, have pharmacological effects such as antioxidant, antimicrobial, anti‐inflammatory, antiatherogenic, anticarcinogenic, antiviral, hypoglycemic, and neuroprotective (Abdel‐Salam et al. 2018; Pennisi et al. 2023; Sánchez‐Gutiérrez et al. 2021; Wang et al. 2008).

Fermented dairy products such as yoghurt are highly susceptible to yeast and mold spoilage during storage. Conventionally, additives such as potassium sorbate have been used to control such spoilage, but with consumer demand for clean‐labeled products, biopreservatives and plant extracts are increasingly being used in milk formulations (Buehler et al. 2018). Olive leaves are also widely utilized in biopreservation due to their rich content of phenolic compounds with well‐documented antimicrobial and antioxidant properties (Sánchez‐Gutiérrez et al. 2021). While previous research has investigated the use of olive leaf extract as a natural preservative to inhibit yeast and mold growth and enhance the functional qualities of yoghurt, limited attention has been given to its potential impact on sensory attributes (Barukčić et al. 2022; Pourghorban et al. 2022; Tavakoli et al. 2018). The strong, characteristic aroma of olive leaf extract often compromises the organoleptic quality of fortified food products, thereby restricting its broader use as a preservative (Korukluoglu et al. 2008). In this context, although the addition of olive leaves and their extracts to yoghurt has been previously explored, to the best of our knowledge, this study is the first to evaluate the application of POLE in the form of an aqueous solution sprayed onto the surface of yoghurt post‐fermentation. This study aimed to investigate the effects of applying aqueous POLE to yoghurt either by surface spraying after fermentation or by incorporation before fermentation at two different concentrations, with a focus on evaluating physicochemical, functional, microbiological, and sensory properties. This approach was designed to assess the potential of POLE as a natural preservative and functional additive while minimizing its negative impact on the sensory quality of yoghurt.

## 2 Materials and Methods

### 2.1 Material

Standardized yoghurt milk was obtained from TUNA Dairy Product, Konya, Türkiye, and POLE was purchased from Zade Vital Pharmaceuticals Inc., Konya, Türkiye. A commercial yoghurt culture comprised of *L. delbrueckii subsp. bulgaricus* and *S. thermophilus* (Chr's Hansen‐Peyma, Istanbul) was used to prepare yoghurt. Aqueous solutions of POLE at 0.4% and 0.6% concentrations were prepared by mixing 40 mL each of sterile pure water in a sterile spray bottle until homogeneous dissolution.

### 2.2 Preparation of Aqueous Solutions of POLE

The flasks containing 40 mL of sterile distilled water were sterilized in an autoclave (121°C for 15–20 min) and cooled to an ambient temperature. POLE was added to sterile water to form 0.4% and 0.6% concentrations and mixed until homogenized. The aqueous leaf extract was freshly prepared before spraying onto the yoghurt surface.

### 2.3 Production of Set Type Yoghurts

Standardized and pasteurized (at 90°C for 10 min) milk was brought to the laboratory from TUNA Dairy Product, Konya, Türkiye, and then heated up to the inoculation temperature of 42°C ± 1°C. Heated milk was divided into 5 experimental parts, and two portions of milk were fortified with 0.4% and 0.6% POLE. The remaining three portions of milk were left to ferment without any addition, one portion as control yoghurt and the others for spraying an aqueous solution of POLE on the surface after incubation. Each portion of prepared milk was inoculated with 2% yoghurt starter culture and then placed in 100 mL sterile plastic cups and incubated at 42°C to the final pH (pH 4.6). For the samples to be prepared by a surface spraying method, after incubation, 2 sprays (≌ 333 μL) of olive leaf extract aqueous solution (0.4% and 0.6% solution) were applied in a sterile cabinet to completely cover the plain yoghurt surface, except for control yoghurt. All experimental yoghurts were kept at 4°C for further analysis throughout 28 days.

### 2.4 Fermentation Kinetics

The pH variations in yoghurt fermentation were recorded every half hour until the pH achieved 4.6. The process kinetics were characterized as the maximum acidification rate (V_max_, 10^−3^ upH/min), the time to reach the maximum acidification rate (T_Vmax_, h), and the time at which was the end of the fermentation (T_pH 4.6_, h) (Gölbaşι et al. 2023).

### 2.5 Physicochemical Analysis

The methods of AOAC (2005) were used to determine total solid (method 990.19), fat (method 989.05), crude ash (method 945.46) and protein (method 991.20) contents of yoghurt samples. These chemical analyses were carried out on the 28th day of refrigerated storage. The pH values of yoghurts were measured with a digital pH meter (pH 315 i/SET, WTW, Weilheim, Germany). The titratable acidity was determined by titration with 0.1 N NaOH and expressed as the percentage of lactic acid (AOAC 2005). Color parameters of samples were detected with a Minolta CR‐400 chromameter (Konica Minolta Sensing Inc. Osaka, Japan), using the CIE scale L* (lightness; 100 = white, 0 = black), a* (redness; ±, red; green), and b* (yellowness; ±, yellow; blue). In the yoghurt samples produced by the surface spraying method, two different measurements were made from the inside and the surface, while in the samples mixed into POLE, it was accepted that the extract was homogeneously mixed, and the measurements were made from the surface of yoghurt. The pH, titratable acidity, and color values of samples were determined on the 1st, 7th, 14th, and 28th day of storage.

### 2.6 Water Holding Capacity (WHC) and Syneresis

To determine the WHC, 5 g yoghurt samples were weighed into falcon tubes and placed in the centrifuge. After centrifugation at 4500 rpm for 30 min at 10°C, the weight of whey was calculated and expressed as the weight of drained whey per 100 g yoghurt (Gölbaşι et al. 2023). For syneresis, 20 g of yoghurt on filter papers (Whatman no: 1) was put on top of a funnel and kept at 4°C for 4 h (Gölbaşι et al. 2023). After that, the drained whey was weighed, and syneresis was calculated by the following formula:

$$\begin{array}{c} \text{Syneresis}\;\left(\%\right)\\ =\left(\text{Weight of whey collected after drainage}/\text{Weight of yoghurt sample}\right)\\ \times 100 \end{array}$$

### 2.7 Texture Profile Analysis (TPA)

Texture profile analysis was performed on a TA‐XT2 Texture Analyzer (Stable Micro Systems, Godalming, England) equipped with a 50 kg load cell as recommended by Göktepe and Akın (2021). Texture measurements were operated at ambient temperature on yoghurt samples removed from refrigerated storage (4°C ± 1°C) immediately prior to testing. The penetration test was implemented on 50 g samples contained in a 100 mL plastic cup using a 36 mm cylindrical probe with a pre‐test speed of 3 mm/s, test speed of 3.3 mm/s, post‐test speed of 3 mm/s, and test distance of 15 mm. Hardness (N), adhesiveness (g.s), springiness, cohesiveness, gumminess (N), and chewiness (N) parameters of yoghurts were determined from the force‐time curve. The test was carried out in three replicates for each yoghurt on the 14th day of storage.

### 2.8 Determination of Antioxidant Activity and Total Phenolic Content (TPC)

For antioxidant activity and TPC analyses, yoghurt sample extracts were prepared using the method of Demirci et al. (2017). These extracts were stored at +4°C prior to use within 24 h.

DPPH (1,1‐diphenyl‐2‐picrylhydrazyl) (Brand‐Williams et al. 1995) and ABTS^●+^ (2,2′‐Azino‐bis‐3‐ethylbenzothiazoline‐6‐sulfonic) (Re et al. 1999) radical scavenging methods were implemented to evaluate the antioxidant capacities of yoghurt samples. DPPH radical scavenging activity was performed at 517 nm and the results were described as % inhibition. ABTS^●+^ radical scavenging capacity was measured at 734 nm and the results were expressed as μM Trolox equivalent/100 g yoghurt. TPC content of yoghurts was analyzed according to the method of McCue and Shetty (2003) by using Folin–Ciocalteu reagent. The results were expressed as mg of GAE/100 g of dry weight. Analyses were performed in triplicate for each repetition, only on day 28 of cold storage.

### 2.9 Microbiological Analysis

The yoghurt sample (10 g) was homogenized with 90 mL of 0.1% sterile Ringer's solution (Merck, Darmstadt, Germany) in a sterile stomacher bag for 1 min and serially diluted in a sterile peptone solution (Ben‐David and Davidson 2014). Enumeration of *S. thermophilus* was performed at 37°C for 48 h on *Streptococcus thermophilus* (ST; Merck, Darmstadt, Germany) agar under microaerophilic conditions (Tharmaraj and Shah 2003). *L. delbrueckii subsp. bulgaricus* colonies were counted on reinforced clostridial agar (RCA; Himedia, Mumbai, India) under anaerobic conditions at 42°C for 48–72 h (Dave and Shah 1996). Yeast extract glucose chloramphenicol (YGC) agar (Merck, Darmstadt, Germany) was used for the enumeration of yeasts and molds at 25°C for 5 days under microaerophilic conditions (Nielsen et al. 2021). Microbiological enumerations were carried out after 1, 7, 14, 2, and 28 days of storage in duplicate using the spread plate method, and colony counts were expressed as log CFU/g.

### 2.10 Sensory Evaluation

Nine trained panelists rated the sensory properties of yoghurt, consisting of appearance, consistency, odor, taste, and overall acceptability, using a nine‐point hedonic scale (1: Very bad—9: Excellent) (Obi et al. 2010). Sensory evaluation was performed on all experimental samples on the 14th day of cold storage.

### 2.11 Statistical Analysis

The data were analyzed by using one‐way ANOVA implemented in MiniTab 7.1 statistical software (Minitab 1991). Statistical significance was assessed at a 95% confidence level, and pairwise comparisons were conducted using Tukey's post hoc test at a significance level of *p* < 0.05.

## 3 Results and Discussion

### 3.1 Acidification Kinetics of Yoghurts During Fermentation

**FIGURE 1:**
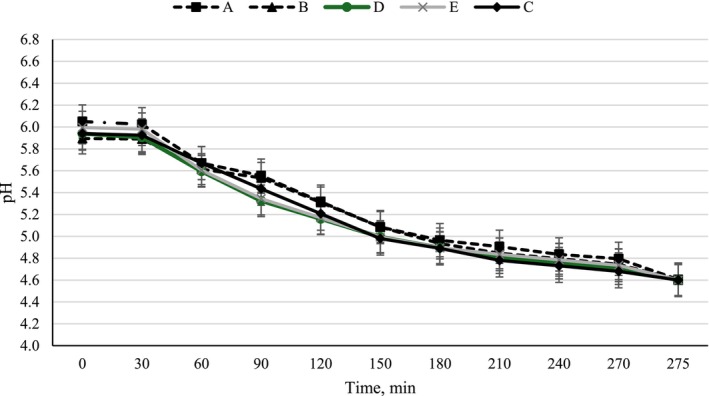
Acidification profiles of yoghurt samples during fermentation. (A: Yoghurt produced by surface spraying of aqueous solutions of olive leaf extract at 0.4% concentration after incubation, B: Yoghurt produced by surface spraying of aqueous solutions of olive leaf extract at 0.6% concentration after incubation, D: Yoghurts produced by adding 0.4% powdered olive leaf extract before incubation, E: Yoghurts produced by adding 0.6% powdered olive leaf extract before incubation, C: Control yoghurt).

Figure 1 shows the pH changes in set‐type yoghurts during fermentation. Considering the shape of the curves, the acidification profiles of all the yoghurt samples exhibited a similar pattern, and it was observed that the addition of POLE did not significantly affect the fermentation times of the set‐type yoghurts. The pH remained almost unchanged for the first 30 min of incubation, which is the adaptation phase of the microorganisms to the substrate and the incubation temperature (Cândido de Souza et al. 2021). After this short adaptation period, pH values showed a decrease with a similar progression in all experimental samples, resulting in an average pH value of 4.6 ± 0.1 at the end of 275 ± 3 min of fermentation. In parallel with our findings, it was stated in previous studies that POLE did not suppress the growth of yoghurt bacteria and therefore did not have a negative effect on the fermentation process (Marhamatizadeh et al. 2013; Peker and Arslan 2017; Pourghorban et al. 2022).

The acidification kinetic results (V_max_, T_Vmax_, T_pH 4.6_) are provided in Table 1. While sample E gave the maximum acidification rate (V_max_) with 12.50 × 10^−3^ upH/min, the control sample exhibited the lowest value with 8.50 × 10^−3^ upH/min. The addition of 0.6% POLE to sample E may have provided the maximum acidification rate during the fermentation of the yoghurt. On the other hand, Georgakouli et al. (2016) investigated the potential role of olive polyphenols in the microflora of yoghurt during fermentation and storage and reported that the addition of olive polyphenols in the first hours of fermentation caused an increase in lactic acid level. This observation was attributed by the researchers to the higher growth of lactic acid bacteria in the samples with olive polyphenols during the fermentation period. The time to reach the maximum acidification rate (T_Vmax_) was 1 h for all experimental samples. Fermentation times (T_pH 4.6_) of all yoghurts varied within a narrow range of 4.53 (yoghurt A) and 4.60 (yoghurt E) h (Table 1). Considering our results, it can be said that the addition of POLE did not have a very significant effect on the T_pH 4.6_ values of the experimental yoghurt, suggesting that POLE may be a favorable material for functional yoghurt production without negatively affecting the fermentation time. Similar outcomes were found in the study of Anuyahong et al. (2020), who stated that the incorporation of anthocyanin‐rich extract from riceberry rice to yoghurt did not alter the fermentation time in comparison to the control group. In contrast to our observations, Barukčić et al. (2022) reported that the addition of olive leaf extract to yoghurt dramatically reduced fermentation times compared to the control.

**TABLE 1:** The acidification kinetic parameters of yoghurts.

Samples^†^	V_max_ (10^−3^ upH/min)	T_Vmax_ (h)	T_pH 4.6_ (h)
A	11.83 ± 1.65^AB^	1.00 ± 0.00^A^	4.60 ± 0.02^A^
B	9.16 ± 0.24^bc^	1.00 ± 0.00^A^	4.57 ± 0.00^AB^
D	10.33 ± 0.47^ABC^	1.00 ± 0.00^A^	4.55 ± 0.00^AB^
E	12.50 ± 0.24^A^	1.00 ± 0.00^A^	4.53 ± 0.00^B^
C	8.50 ± 0.24^C^	1.00 ± 0.00^A^	4.53 ± 0.00^B^

*Note:* Results are expressed as mean ± standard deviation (*n* = 2). ^A–C^Different letters in the same column indicate statistically significant differences between samples (*p* < 0.05), determined by one‐way ANOVA followed by Tukey's post hoc test. (^†^A: yoghurt produced by surface spraying of aqueous solutions of olive leaf extract at 0.4% concentration after incubation, B: yoghurt produced by surface spraying of aqueous solutions of olive leaf extract at 0.6% concentration after incubation, D: yoghurts produced by adding 0.4% powdered olive leaf extract before incubation, E: yoghurts produced by adding 0.6% powdered olive leaf extract before incubation, C: control yoghurt).

Abbreviations: T_max_, time to reach V_max_; T_pH 4.6_, time to reach pH 4.6; V_max_, maximum acidification rate.

### 3.2 Physicochemical Properties of Yoghurts

**FIGURE 2:**
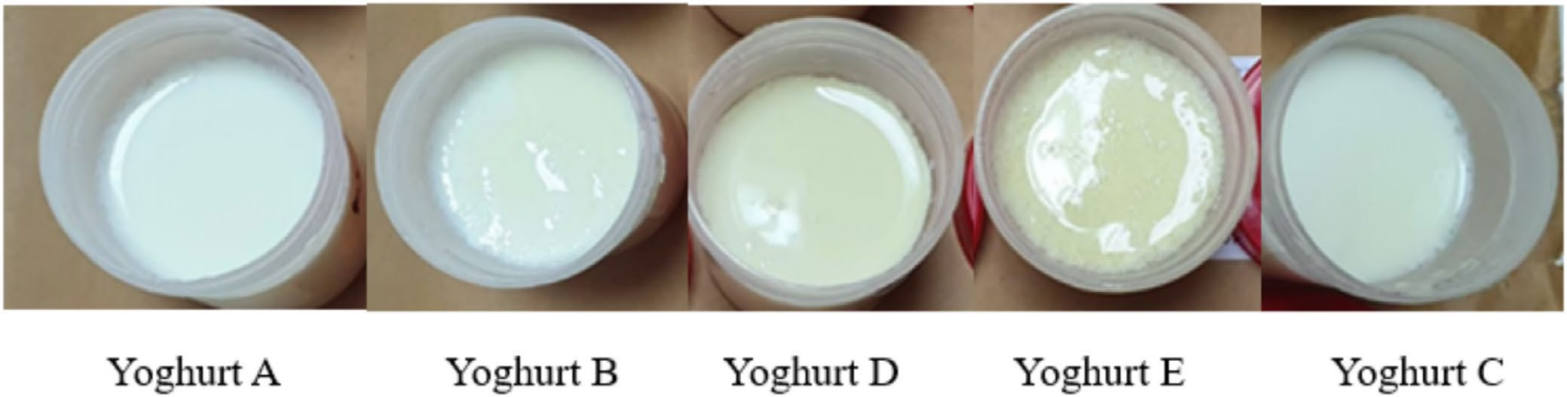
The photograph of yoghurt samples. (A: Yoghurt produced by surface spraying of aqueous solutions of olive leaf extract at 0.4% concentration after incubation, B: Yoghurt produced by surface spraying of aqueous solutions of olive leaf extract at 0.6% concentration after incubation, D: Yoghurts produced by adding 0.4% powdered olive leaf extract before incubation, E: Yoghurts produced by adding 0.6% powdered olive leaf extract before incubation, C: Control yoghurt).

The photographs of yoghurt samples are shown in Figure 2. As illustrated, visual differences among the samples are noticeable, particularly in surface color and homogeneity, depending on both the method of POLE application (before or after incubation) and the extract concentration. These visual variations may be attributed to the presence of phenolic compounds in olive leaf extract, which can interact with the yoghurt matrix and influence its surface appearance (Cho et al. 2020). The results of total solids, fat, protein, and ash in all experimental yoghurts on the 28th day of storage are given in Table 2. The total solids, fat, protein, and ash contents for samples ranged from 13.99% to 14.70%, 0.85% to 1.18%, 3.67% to 3.93%, and 1.16% to 1.33%, respectively. There was no statistical difference between the samples for each parameter evaluated (*p* < 0.05). Yadav et al. (2018) reported similar findings, where no significant difference was observed between the chemical compositions (total solids, fat, ash, and protein contents) of yoghurt enriched with grape seed extract and plain yoghurt.

**TABLE 2:** The total solids, fat, protein, and ash contents of yoghurts on day 28 of storage.

Samples^†^	Total solid (%)	Fat (%)	Protein (%)	Ash (%)
A	13.99 ± 0.13^A^	1.18 ± 0.28^A^	3.82 ± 0.02^A^	1.16 ± 0.23^A^
B	14.20 ± 0.25^A^	0.91 ± 0.04^A^	3.67 ± 0.00^A^	1.16 ± 0.23^A^
D	14.59 ± 0.12^A^	0.89 ± 0.08^A^	3.81 ± 0.21^A^	1.33 ± 0.00^A^
E	14.70 ± 0.66^A^	0.86 ± 0.03^A^	3.76 ± 0.08^A^	1.17 ± 0.70^A^
C	14.30 ± 0.25^A^	0.85 ± 0.09^A^	3.93 ± 0.12^A^	1.17 ± 0.23^A^

*Note:* Results are expressed as mean ± standard deviation (*n* = 2). All values in the same column share the same letter, indicating no statistically significant differences among samples (*p* > 0.05), as determined by one‐way ANOVA followed by Tukey's post hoc test. (^†^A: yoghurt produced by surface spraying of aqueous solutions of olive leaf extract at 0.4% concentration after incubation, B: yoghurt produced by surface spraying of aqueous solutions of olive leaf extract at 0.6% concentration after incubation, D: yoghurts produced by adding 0.4% powdered olive leaf extract before incubation, E: yoghurts produced by adding 0.6% powdered olive leaf extract before incubation, C: control yoghurt).

**FIGURE 3:**
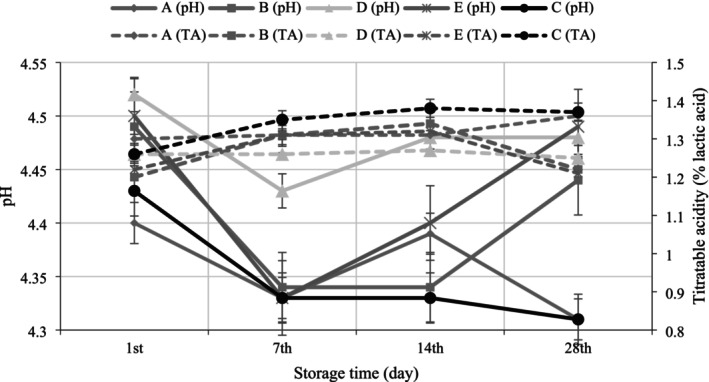
Changes in pH and titratable acidity of yoghurt samples during storage. (A: Yoghurt produced by surface spraying of aqueous solutions of olive leaf extract at 0.4% concentration after incubation, B: Yoghurt produced by surface spraying of aqueous solutions of olive leaf extract at 0.6% concentration after incubation, D: Yoghurts produced by adding 0.4% powdered olive leaf extract before incubation, E: Yoghurts produced by adding 0.6% powdered olive leaf extract before incubation, C: Control yoghurt).

The results for pH value and titratable acidity of yoghurt samples during 28 days of storage are presented in Figure 3. The addition of POLE in yoghurt before incubation and the spraying of different concentrations of aqueous solutions of POLE on the surface of yoghurt after incubation gave statistically similar results in the pH values with the opposite trend of titratable acidity, except on the 28th day of storage. On the 28th day of storage, yoghurt A and the control group had the lowest pH value with pH 4.31, while these samples had the highest titratable acidity among the samples with 1.36% and 1.37%, respectively, in accordance with the pH results. Our findings demonstrated that the POLE treatments in yoghurt did not affect the pH and titratable acidity values of the experimental samples except at the end of storage. The lack of significant changes in pH and titratable acidity values in POLE‐treated yoghurt samples, except at the end of the storage period, may be attributed to the limited buffering capacity and low reactivity of phenolic compounds at the concentrations used (Tosif et al. 2021). This agreed with Tarchi et al. (2025) and El‐Messery et al. (2021), who noted no significant changes in pH and titratable acidity with the incorporation of olive leaf extract in yoghurt. Similar findings were reported by Ferreira and Santos (2023) who observed that phenolic extracts derived from agro‐industrial by‐products such as chestnut shells, pomegranate peels, and grape seeds had no significant effect on the pH of yoghurt samples. On the other hand, the pH of the control group decreased from 4.43 to 4.31 at the end of the storage time as compared to the first day (*p* = 0.033), but there was no significant decrease in the pH value of the other samples (*p* > 0.05). This is consistent with our microbiological analysis result that neither the addition of POLE before incubation nor the spraying of aqueous solutions of POLE on the surface of the yoghurt after incubation had a significant effect on the activities of the yoghurt starter bacteria during storage.

Table 3 shows the results from internal and surface color measurement of experimental yoghurts. Moreover, the color parameters of yoghurts A and B produced by surface spraying of aqueous solutions of POLE at different concentrations after incubation were recorded by taking measurements from the surface and compared with the control group. In samples D and E, it was assumed that POLE was homogeneously mixed into yoghurt milk during production, and surface measurements were not performed separately. Adding POLE to yoghurt caused a statistically significant decrease in brightness (L*) in yoghurts D and E; this darkening was greatest in sample E with increasing POLE concentration on the first day of storage (Table 3). It has been reported in previous studies that natural olive polyphenol extracts have a yellow to dark brown color (Georgakouli et al. 2016). Furthermore, there is no statistically significant difference between the internal L* values of samples A, B, and C. This may be related to the fact that yoghurt A and B were produced by surface spraying of aqueous solutions of POLE after fermentation and contained low concentrations of POLE. L* values of internal measurements for all samples were not significantly altered during 28 days of storage (*p* > 0.05). Similarly, Tavakoli et al. (2018) noted that plain yoghurt had higher brightness than yoghurt containing olive leaf extract. Besides, Barukčić et al. (2022) observed that the difference in color of samples in yoghurt enriched with olive leaf extract increased with the amount of extract added, but there was no significant difference in color values between samples according to storage time. However, brightness was significantly lower in samples A and B compared to the control group in accordance with surface measurement results on the first day of storage (*p* = 0.021) (Table 3). This difference in L* values on the surface was close to being statistically insignificant as storage time progressed. The reason for the lightening of the surface color of A and B due to storage may be the penetration of the extract sprayed on the surface into the yoghurt over time.

**TABLE 3:** Color properties of yoghurts with internal and surface measurement results.

	Samples^†^	Storage time (days)			
		1	7	14	28
Internal color parameters					
L*	A	93.80 ± 0.18^A,a^	93.56 ± 0.23^A,a^	92.77 ± 0.11^A,b^	93.41 ± 0.13^A,ab^
	B	93.37 ± 0.21^A,a^	92.70 ± 0.69^A,a^	93.22 ± 0.33^A,a^	92.75 ± 0.41^A,a^
	D	90.61 ± 0.54^B,a^	90.18 ± 0.15^B,a^	90.09 ± 0.04^B,a^	89.24 ± 0.64^B,a^
	E	89.93 ± 0.02^B,a^	90.37 ± 0.01^B,a^	89.25 ± 0.81^B,a^	89.92 ± 0.43^B,a^
	C	93.19 ± 0.11^A,a^	93.45 ± 0.47^A,a^	93.25 ± 0.64^A,a^	92.69 ± 0.01^A,a^
a*	A	−3.01 ± 0.09^bc,a^	−3.21 ± 0.01^B,ab^	−3.35 ± 0.03^B,b^	−3.85 ± 0.08^C,c^
	B	−2.64 ± 0.15^A,a^	−2.78 ± 0.14^A,ab^	−3.15 ± 0.07^AB,bc^	−3.34 ± 0.11^B,c^
	D	−3.14 ± 0.01^C,ab^	−2.92 ± 0.13^AB,a^	−3.11 ± 0.01^AB,ab^	−3.27 ± 0.06^B,b^
	E	−3.26 ± 0.06^C,a^	−3.15 ± 0.01^AB,a^	−3.22 ± 0.06^AB,a^	−3.21 ± 0.08^AB,a^
	C	−2.77 ± 0.07^AB,a^	−3.10 ± 0.06^AB,a^	−3.00 ± 0.13^A,a^	−2.89 ± 0.07^A,a^
b*	A	2.44 ± 0.41^B,bc^	3.11 ± 0.06^B,ab^	2.18 ± 0.12^B,c^	3.66 ± 0.00^B,a^
	B	1.57 ± 0.36^B,a^	1.79 ± 0.71^B,a^	2.68 ± 0.09^B,a^	2.69 ± 0.99^B,a^
	D	8.13 ± 0.78^A,a^	9.07 ± 0.16^A,a^	8.42 ± 0.95^A,a^	8.80 ± 0.47^A,a^
	E	8.74 ± 0.77^A,a^	9.57 ± 0.37^A,a^	8.58 ± 0.13^A,a^	9.75 ± 0.13^A,a^
	C	1.90 ± 0.06^B,b^	3.34 ± 0.38^B,a^	2.43 ± 0.49^B,ab^	2.33 ± 0.12^B,ab^
Surface color parameters					
L*	A	82.29 ± 0.05^B,b^	91.77 ± 2.82^Aa^	93.73 ± 0.21^A,a^	93.71 ± 0.25^A,a^
	B	87.46 ± 3.11^AB,a^	92.67 ± 1.19^A,a^	93.18 ± 0.49^A,a^	93.34 ± 0.23^AB,a^
	C	93.19 ± 0.11^A,a^	93.45 ± 0.47^A,a^	93.25 ± 0.64^A,a^	92.69 ± 0.01^B,a^
a*	A	−3.32 ± 0.15^A,a^	−3.14 ± 0.04^A,a^	−3.44 ± 0.24^A,a^	−3.55 ± 0.23^A,a^
	B	−3.21 ± 0.27^A,a^	−3.29 ± 0.28^A,a^	−3.40 ± 0.12^A,a^	−3.45 ± 0.12^A,a^
	C	−2.77 ± 0.07^A,a^	−3.10 ± 0.06^A,a^	−3.00 ± 0.13^A,a^	−2.89 ± 0.07^A,a^
b*	A	5.10 ± 0.49^A,a^	4.34 ± 0.35^A,ab^	2.94 ± 0.63^A,b^	3.80 ± 0.11^A,ab^
	B	5.13 ± 0.04^A,a^	4.01 ± 0.97^A,a^	3.81 ± 0.08^A,a^	4.24 ± 0.25^A,a^
	C	1.90 ± 0.06^B,b^	3.34 ± 0.38^A,a^	2.43 ± 0.49^A,ab^	2.33 ± 0.12^B,ab^

*Note:* Results are expressed as mean ± standard deviation (*n* = 2). ^A,B^Different upper‐case letters in the same column show statistical differences between samples (*p* < 0.05), determined by one‐way ANOVA followed by Tukey's post hoc test. ^a,b^Different lower‐case letters in the same row show statistical difference between storage periods (*p* < 0.05), determined by one‐way ANOVA followed by Tukey's post hoc test. (^†^A: yoghurt produced by surface spraying of aqueous solutions of olive leaf extract at 0.4% concentration after incubation, B: yoghurt produced by surface spraying of aqueous solutions of olive leaf extract at 0.6% concentration after incubation, D: yoghurts produced by adding 0.4% powdered olive leaf extract before incubation, E: yoghurts produced by adding 0.6% powdered olive leaf extract before incubation, C: control yoghurt).

As shown in Table 3, yoghurt E exhibited the lowest internal a* value on day 1 (*p* = 0.004), indicating the strongest green tone. However, no statistically significant variation was observed in a* values throughout the storage period (*p* = 0.379). On the other hand, the highest yellowness (b* value) was recorded in the same sample (E) with a value of 8.74. The combination of the most intense greenness and yellowness in yoghurt E may be attributed to the intrinsic color properties of the POLE, which contains chlorophylls and various phenolic compounds known to contribute to greenish and yellowish tones in food matrices (Georgakouli et al. 2016). According to the surface color parameters, the control group exhibited the highest a* value, indicating the lowest degree of greenness among all samples. However, the difference in a* values was not statistically significant (*p* = 0.109), suggesting that POLE treatments did not cause a meaningful change in surface greenness. In addition, yoghurts A and B exhibited significantly higher yellowness values of the surface compared to the control group because of the original color of POLE (*p* = 0.002). The b* values obtained from internal measurement increased in all samples at the end of storage compared to the first day, while the b* values of the surface in yoghurts A and B showed a decrease during storage. This may be related to the penetration of the sprayed extract into the yoghurt matrix. Similarly, the trends observed in our a* and b* values were in agreement with those reported by Cho et al. (2017), who found comparable changes in color parameters when yoghurt was formulated with green olive powder. These findings collectively suggest that the use of olive‐derived compounds can influence yoghurt color dynamics in a manner dependent on application method and storage conditions.

### 3.3 WHC and Syneresis of Yoghurts

The WHC and syneresis values of all experimental samples and the changes during 28 days of cold storage are shown in Table 4. The WHC of yoghurts varied from 46.93% to 50.65% on the first day of storage, and POLE addition and spraying the yoghurt surface with aqueous solutions of POLE had no significant effect on the WHC (*p* = 0.260). Although the WHC of all experimental yoghurts increased slightly on the last day of storage compared to the first day, this increment was not statistically significant (*p* = 0.062). This slight increase until the end of storage may be attributed to the formation of a protein network with a higher water‐binding capacity by the possible interaction between olive polyphenols and milk proteins during storage (Charlton et al. 2002; Oliveira et al. 2001). Opposite observations were reported by Tarchi et al. (2025), who found that WHC values increased with higher OLE concentrations compared to the control throughout the storage period.

**TABLE 4:** Effect of different treatments of olive leaf extract on water holding capacity (WHC) and syneresis of yoghurts.

Parameters	Samples^†^	Storage time (days)			
		1	7	14	28
WHC (%)	A	49.25 ± 2.61^A,a^	50.90 ± 1.55^A,a^	50.95 ± 0.77^A,a^	50.00 ± 1.41^A,a^
	B	49.65 ± 1.91^A,a^	48.11 ± 1.27^AB,a^	45.58 ± 1.77^A,a^	51.29 ± 0.54^A,a^
	D	50.50 ± 0.99^A,a^	48.16 ± 1.75^AB,a^	48.61 ± 0.85^A,a^	50.55 ± 1.34^A,a^
	E	46.93 ± 0.83^A,ab^	43.23 ± 0.57^B,b^	46.60 ± 1.41^A,ab^	49.25 ± 0.78^A,a^
	C	50.65 ± 0.35^A,a^	47.27 ± 1.34^AB,a^	54.43 ± 5.41^A,a^	53.54 ± 1.06^A,a^
Syneresis (%)	A	26.59 ± 4.29^A,a^	26.50 ± 1.56^A,a^	21.74 ± 0.91^AB,a^	21.77 ± 3.29^A,a^
	B	26.75 ± 1.55^A,a^	27.24 ± 0.10^A,a^	21.89 ± 4.05^AB,a^	26.13 ± 1.75^A,a^
	D	25.32 ± 4.41^A,a^	29.77 ± 3.44^A,a^	26.79 ± 0.26^AB,a^	26.11 ± 1.05^A,a^
	E	24.87 ± 2.36^A,a^	29.14 ± 0.90^A,a^	29.70 ± 0.98^A,a^	27.55 ± 1.20^A,a^
	C	28.53 ± 0.86^A,a^	25.45 ± 0.66^A,a^	18.51 ± 4.48^B,a^	20.21 ± 4.29^A,a^

*Note:* Results are expressed as mean ± standard deviation (*n* = 2). ^A,B^Different upper‐case letters in the same column show statistical differences between samples (*p* < 0.05), determined by one‐way ANOVA followed by Tukey's post hoc test. ^a,b^Different lower‐case letters in the same row show statistical difference between storage periods (*p* < 0.05), determined by one‐way ANOVA followed by Tukey's post hoc test. (^†^A: yoghurt produced by surface spraying of aqueous solutions of olive leaf extract at 0.4% concentration after incubation, B: yoghurt produced by surface spraying of aqueous solutions of olive leaf extract at 0.6% concentration after incubation, D: yoghurts produced by adding 0.4% powdered olive leaf extract before incubation, E: yoghurts produced by adding 0.6% powdered olive leaf extract before incubation, C: control yoghurt).

As seen in Table 4, all treatments did not significantly change the syneresis value of yoghurts. In addition, the syneresis of the samples showed a decreasing trend during the storage period, and this diminishment was not statistically significant (*p* > 0.05). In agreement with our study, Barukčić et al. (2022) stated that the incorporation of olive leaf extract into yoghurt and storage time did not significantly affect WHC and syneresis levels, wherein the determined WHC and syneresis values were 58.20% and 37.60% in control and 56.00%–60.50% and 38.00%–40.00% in the yoghurt added olive leaf extract (1.5%, 3%, and 5% v/v), respectively. Our results for WHC were slightly lower compared to results obtained by Barukčić et al. (2022), which may have resulted from the difference in total solid content, starter culture activity, incubation time/temperatures, and cooling/storage conditions (Bierzuńska et al. 2019). In contrast to our results, El‐Messery et al. (2021) and Tavakoli et al. (2018) found a notable decrease in syneresis upon the addition of encapsulated OLE to yoghurt. This reduction in syneresis could be linked to the increase in total solids within the yoghurt and the interaction between the polyphenols in OLE and the milk proteins (Zoidou et al. 2017). Similarly, earlier studies reported that supplementation with *Nelumbo nucifera* leaf extract (Joung et al. 2016) and moringa leaf powder extract (Zhang et al. 2019) resulted in increased WHC and reduced syneresis in yoghurt, compared to plain yoghurt, by stabilizing the gel matrix.

### 3.4 Textural Characteristics of Yoghurts

Table 5 shows the textural properties (hardness, adhesiveness, springiness, cohesiveness, gumminess, and chewiness) of all yoghurts on the 14th day of storage. Hardness values of experimental samples varied between 3.46 and 4.18 N, with the highest value observed in the control group and the lowest in yoghurt D. Yoghurts produced by spraying aqueous solutions of POLE on the surface after incubation (yoghurts A and B) gave results closer to the hardness of the control yoghurt. However, these differences in hardness results were not statistically significant (*p* = 0.098). The reduction in hardness with the addition of POLE in yoghurt could be due to increased water in the gel system, which probably led to a softer gel network of yoghurt. Furthermore, the higher hardness exhibited by the control yoghurt was most likely due to the higher protein rearrangement in this yoghurt (Mohamed Ahmed et al. 2021). In general, the addition of olive leaf extract increases cohesion by strengthening the protein system through polyphenol‐protein interactions. However, these interactions may disrupt the natural structure of proteins, leading to a decrease in hardness (Tarchi et al. 2025). Similar to our results, previous studies indicated that incorporations of yoghurts with plant extracts such as olive, onion, citrus, and garlic (Michael et al. 2010) and the *Pleurotus ostreatus* aqueous extract (Vital et al. 2015) reduce the hardness of yoghurts compared to plain yoghurt. Contrary to our findings, Tarchi et al. (2025) reported that the hardness of yoghurt samples increased with both higher concentrations of olive leaf extract and extended storage time.

**TABLE 5:** The textural parameters of yoghurts on the 14th day of storage.

Samples^†^	Parameters					
	Hardness (N)	Adhesiveness (g.s)	Springiness	Cohesiveness	Gumminess (N)	Chewiness (N)
A	3.92 ± 0.02^A^	334.88 ± 45.75^BC^	0.99 ± 0.01^A^	0.39 ± 0.00^A^	1.52 ± 0.01^AB^	1.50 ± 0.02^AB^
B	3.81 ± 0.43^A^	298.92 ± 13.33^AB^	1.00 ± 0.00^A^	0.40 ± 0.01^A^	1.51 ± 0.12^AB^	1.51 ± 0.12^AB^
D	3.46 ± 0.15^A^	241.89 ± 1.35^A^	0.98 ± 0.01^A^	0.39 ± 0.01^A^	1.36 ± 0.03^B^	1.34 ± 0.02^B^
E	3.61 ± 0.07^A^	270.13 ± 0.06^AB^	0.99 ± 0.02^A^	0.40 ± 0.03^A^	1.45 ± 0.10^B^	1.43 ± 0.13^B^
C	4.18 ± 0.10^A^	419.64 ± 8.96^C^	0.99 ± 0.01^A^	0.42 ± 0.02^A^	1.76 ± 0.02^A^	1.75 ± 0.04^A^

*Note:* Results are expressed as mean ± standard deviation (*n* = 2). ^A–C^Different letters in the same column indicate statistically significant differences between samples (*p* < 0.05), determined by one‐way ANOVA followed by Tukey's post hoc test. (^†^A: yoghurt produced by surface spraying of aqueous solutions of olive leaf extract at 0.4% concentration after incubation, B: yoghurt produced by surface spraying of aqueous solutions of olive leaf extract at 0.6% concentration after incubation, D: yoghurts produced by adding 0.4% powdered olive leaf extract before incubation, E: yoghurts produced by adding 0.6% powdered olive leaf extract before incubation, C: control yoghurt).

The adhesiveness values were determined to be higher in yoghurts produced by adding POLE than in control. In this context, it can be considered that POLE improves the adhesiveness value by forming different intermolecular interactions. Previous results in the literature demonstrated that plant extracts could affect yoghurt structure (Ahmed et al. 2024; Domagala et al. 2005). In addition, Almusallam et al. (2021) reported that the incorporation of date palm spikelet extracts advanced the adhesiveness of yoghurt. The increase in adhesiveness with the addition of plant extracts probably resulted from the high‐water absorption ability of the protein matrix and the high protein content of the yoghurt composition (Costa et al. 2015).

The springiness and cohesiveness values of yoghurts ranged from 0.98 to 1.00 and 0.39 to 0.42, respectively (Table 5). As can be seen from the results varying within a very narrow range, POLE treatment had no significant effect on these parameters (*p* > 0.05). Similarly, Fan et al. (2024) discovered that incorporating water‐soluble extract from *Semen Ziziphi Spinosae* into yoghurt had no significant effects regarding springiness and cohesiveness. In contrast to our results, the increase in cohesiveness and springiness of yoghurt enriched with argel leaf extract during storage was attributed by Mohamed Ahmed et al. (2021) to the decrease in pH and metabolic activity of lactic acid bacteria leading to structural shrinkage and reorganization. In addition, the aforementioned study suggested that the interaction between argel leaf extract phenolic compounds and yoghurt proteins may lead to protein rearrangement during storage, thus stabilizing casein networks and improving yoghurt consistency.

Gumminess and chewiness are directly correlated with each other (Azari‐Anpar et al. 2017), and POLE treatments significantly affected these texture parameters in the present study. The addition of POLE into yoghurt during production and the spraying of POLE aqueous extract on the yoghurt's surface caused a significant decrease in the gumminess and chewiness values of the samples compared to the control. The observed reduction in gumminess and chewiness in POLE‐treated samples is likely related to the reduction in hardness, which has a central role in these textural parameters, as they are directly related. Phenolic compounds present in POLE may interfere with the formation and strength of the protein gel network in yoghurt by binding to casein micelles or altering protein–protein interactions, resulting in a softer, less cohesive structure (Muntaha et al. 2025). At the same time, the increase in adhesiveness noted in POLE‐added samples could be attributed to changes in water‐binding capacity and surface interactions within the yoghurt matrix, possibly caused by phenolic‐protein and phenolic‐polysaccharide interactions (Li et al. 2025). Consistent with these findings, Kaur and Riar (2020) reported that yoghurts fortified with β‐glucan showed lower gumminess than the control yoghurt. In addition, since the aqueous extract was sprayed on the yoghurt surface in samples A and B, these parameters were close to the control sample. On the other hand, it was observed that the POLE concentration used in the different treatments had no significant effect on all texture parameter values. Gumminess is a defect that has an undesirable impact on the appearance and texture of yoghurt and is perceived by the consumer as having a viscous (slimy) feel (Dar and Light 2014). However, in contradiction with our findings, previous studies have reported that the addition of *Aloe vera* *foliar* gel (Azari‐Anpar et al. 2017) and flaxseed (Mousavi et al. 2019) increases gumminess in yoghurt.

### 3.5 Assessment of the TPC and Antioxidant Activity of *Yoghurts*

**FIGURE 4:**
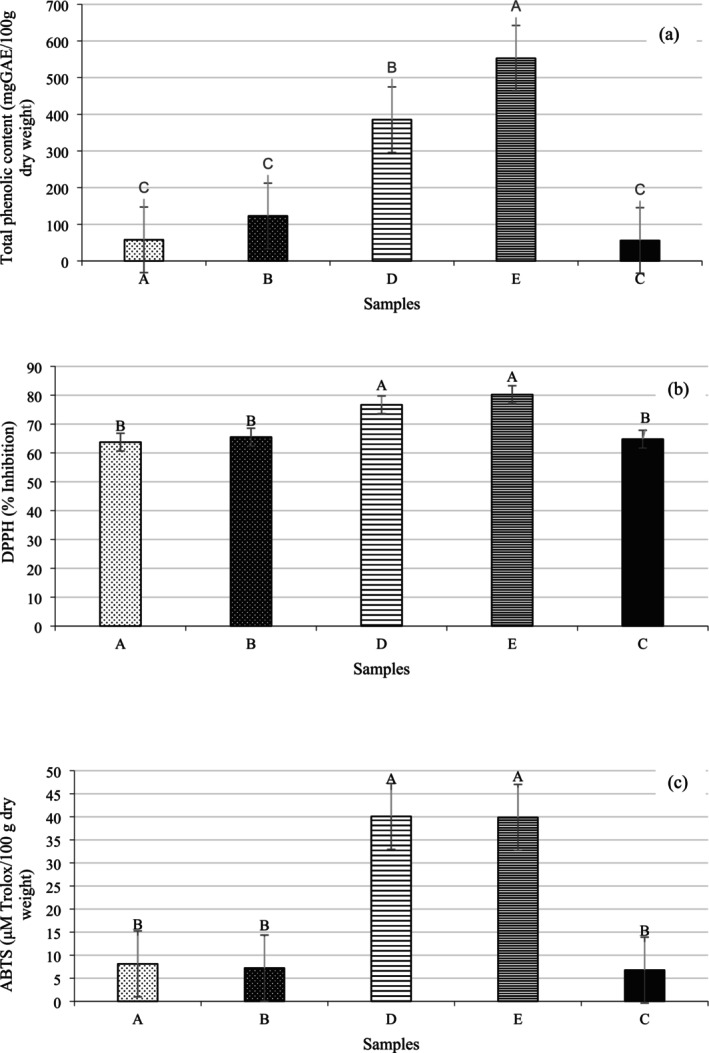
Total phenolic content (a), DPPH (b), and ABTS (c) radical scavenging potentials of the yoghurts on the 28th day of storage. (A: Yoghurt produced by surface spraying of aqueous solutions of olive leaf extract at 0.4% concentration after incubation, B: Yoghurt produced by surface spraying of aqueous solutions of olive leaf extract at 0.6% concentration after incubation, D: Yoghurts produced by adding 0.4% powdered olive leaf extract before incubation, E: Yoghurts produced by adding 0.6% powdered olive leaf extract before incubation, C: Control yoghurt).

The TPC and antioxidant activity (assessed by DPPH and ABTS radical scavenging activities) of all experimental yoghurts on the 28th day of storage are shown in Figure 4. As seen in Figure 4a, the TPC of samples A and B, in which POLE aqueous extract was sprayed on the yoghurt surface, gave values very close to the control yoghurt, whereas the TPC of the yoghurt to which POLE was added before fermentation was dramatically higher than the other samples. Additionally, increasing the POLE concentration significantly enhanced the TPC in yoghurts (*p* = 0.000). Yoghurt E had the highest TPC (552.80 mg GAE/100 g dry weight), while the lowest TPC (55.97 mg GAE/100 g dry weight) was recorded for plain yoghurt. The increased TPC with the addition of plant extracts to yoghurt is most likely due to the phytochemical content of the plant extracts and microbial metabolic activity (Hamed et al. 2021). In parallel with our results, Pourghorban et al. (2022) reported a positive correlation between the concentration of olive leaf powder and its extract added to yoghurt and TPC. Consistently, Tarchi et al. (2025) also found that the enrichment of yoghurt with olive leaf extract resulted in a proportional increase in TPC, depending on the amount of extract incorporated.

The literature shows that olive leaves' rich polyphenol profile can potentially be used as a source of natural antioxidants (Refai et al. 2024). Therefore, as expected, the addition of POLE improved the antioxidant activities of yoghurts. Figure 4b, shows that the results of the antioxidant tests, as obtained in the DPPH and ABTS assays, were compatible for all the yoghurt samples. Also, a positive correlation was observed between TPC and antioxidant activity, and this result is consistent with the findings of many research groups (Tami et al. 2022; Yildiz and Ozcan 2019). In line with this, Martín‐García et al. (2022) showed that the presence of several phenolic compounds, such as oleoside, secologanoside, tyrosol and its glucoside, elenolic acid glucoside, luteolin glucoside, and oleuropein derivatives, was strongly correlated with the antioxidant capacity of olive leaf extract as determined by DPPH radical scavenging activity. According to the in vitro DPPH assay, the antioxidant activity of the control, yoghurt A, and yoghurt B were 64.77%, 63.73%, and 65.49% inhibition, respectively. These closely related values did not differ significantly from one another. On the other hand, the scavenging activities of DPPH radicals increased significantly with the addition of POLE before fermentation (*p* = 0.002). Besides, yoghurt produced by adding 0.6% POLE (sample E) gave the highest radical scavenging activities with 80.21% inhibition.

The ABTS radical scavenging activity in control yoghurt and in yoghurts produced by a surface spraying method with aqueous solutions of POLE after fermentation ranged from 6.76 to 8.11 μM Trolox/100 g on a dry weight basis, and the differences between these yoghurts were not statistically significant (Figure 4c). These values suggest that the surface application of POLE had a limited impact on enhancing the antioxidant capacity of yoghurt. In contrast, the direct incorporation of 0.4% and 0.6% POLE into milk prior to fermentation led to a marked increase in ABTS scavenging activity, reaching up to 5.92‐ and 5.90‐fold higher values, respectively, compared to plain yoghurt. This pronounced enhancement may be attributed not only to the improved integration and stability of phenolic compounds during the fermentation process, but also to the significantly higher concentration of POLE present in the bulk of the product when added before fermentation, compared to the small amount applied on the surface in the spraying method. The direct addition allows for better dispersion and interaction of phenolics with milk proteins, leading to their better retention and antioxidant functionality within the yoghurt matrix. Similar trends demonstrating increased antioxidant activity with rising olive leaf extract concentrations in yoghurt formulations have also been reported by Cho et al. (2020) and Peker and Arslan (2017), supporting the findings of the present study.

### 3.6 Microbiological Characteristics of Yoghurts

Table 6 shows *S. thermophilus* and *L. delbrueckii* subsp. *bulgaricus* counts in yoghurt samples during 4 weeks of cold storage. Monitoring bacterial viability in yoghurt containing POLE was important as olive leaf extract is an antimicrobial agent (Sudjana et al. 2009). Neither the addition of POLE to the yoghurt nor the spraying of POLE aqueous extract on the yoghurt surface caused any change in the populations of yoghurt bacteria compared to the control during storage. In parallel with our findings, Pourghorban et al. (2022) reported that the addition of different concentrations of olive leaf powder and its extract during pre‐ and post‐fermentation had no effect on the viable counts of yoghurt bacteria. Only on the 7th day of storage did the number of *S. thermophilus* significantly differ between the samples, with the lowest count of 8.41 log CFU/g in control yoghurt (Table 6). As can be seen from the results, the addition of POLE and storage time had no negative effect on the survivability of neither *S. thermophilus* nor *L. delbrueckii* subsp. *bulgaricus*, and all yoghurts had similar bacterial counts (> 8 log CFU/g). Similarly, Barukčić et al. (2022) found that the incorporation of olive leaf extract into yoghurt had no noticeable effect on the viable cell count of the yoghurt starter culture. Furthermore, Zoidou et al. (2014) revealed that the addition of oleuropein did not cause significant differences in the total microflora in milk and yoghurts compared to the sample without oleuropein. According to the yeast and mold microbial analysis, no mold and yeast growth responsible for significant economic losses was detected in any of the experimental yoghurts during 28 days of storage. The storage period was extended outside the routine analysis program, and the samples continued to be stored in the refrigerator for observation. The presence of yeast and mold was first observed in the control sample after 42 days of storage, while no visible change was identified in the other yoghurts.

**TABLE 6:** The viable counts of *S. thermophilus* and *L. delbrueckii* subsp. *bulgaricus* in yoghurts during storage.

	Samples^†^	Storage time (days)			
		1	7	14	28
*S. thermophilus* (log CFU/g)	A	8.84 ± 0.15^A,a^	8.78 ± 0.05^AB,a^	8.83 ± 0.19^A,a^	8.81 ± 0.07^A,a^
	B	8.91 ± 0.10^A,a^	8.69 ± 0.09^AB,ab^	8.82 ± 0.04^A,a^	8.47 ± 0.07^A,b^
	D	8.55 ± 0.59^A,a^	8.58 ± 0.08^AB,a^	8.84 ± 0.40^A,a^	8.61 ± 0.03^A,a^
	E	8.47 ± 0.43^A,a^	8.84 ± 0.01^A,a^	8.64 ± 0.10^A,a^	8.62 ± 0.18^A,a^
	C	8.70 ± 0.20^A,a^	8.41 ± 0.17^B,a^	8.44 ± 0.38^A,a^	8.75 ± 0.03^A,a^
*L. delbrueckii* subsp. *bulgaricus* (log CFU/g)	A	8.51 ± 0.33^A,a^	8.72 ± 0.08^A,a^	8.42 ± 0.25^A,a^	8.15 ± 0.09^A,a^
	B	8.80 ± 0.21^A,a^	8.98 ± 0.30^A,a^	8.18 ± 0.10^A,a^	8.18 ± 0.14^A,a^
	D	8.44 ± 0.43^A,a^	8.39 ± 0.14^A,a^	8.14 ± 0.11^A,a^	8.26 ± 0.02^A,a^
	E	8.54 ± 0.36^A,a^	8.61 ± 0.17^A,a^	8.41 ± 0.07^A,a^	8.38 ± 0.11^A,a^
	C	8.75 ± 0.04^A,a^	8.40 ± 0.13^A,a^	8.21 ± 0.25^A,a^	8.26 ± 0.06^A,a^

*Note:* Results are expressed as mean ± standard deviation (*n* = 2). ^A,B^Different upper‐case letters in the same column show statistical differences between samples (*p* < 0.05), determined by one‐way ANOVA followed by Tukey's post hoc test. ^a,b^Different lower‐case letters in the same row show statistical difference between storage periods (*p* < 0.05), determined by one‐way ANOVA followed by Tukey's post hoc test. (^†^A: yoghurt produced by surface spraying of aqueous solutions of olive leaf extract at 0.4% concentration after incubation B: yoghurt produced by surface spraying of aqueous solutions of olive leaf extract at 0.6% concentration after incubation, D: yoghurts produced by adding 0.4% powdered olive leaf extract before incubation E: yoghurts produced by adding 0.6% powdered olive leaf extract before incubation, C: control yoghurt).

### 3.7 Sensory Properties of Yoghurts

**FIGURE 5:**
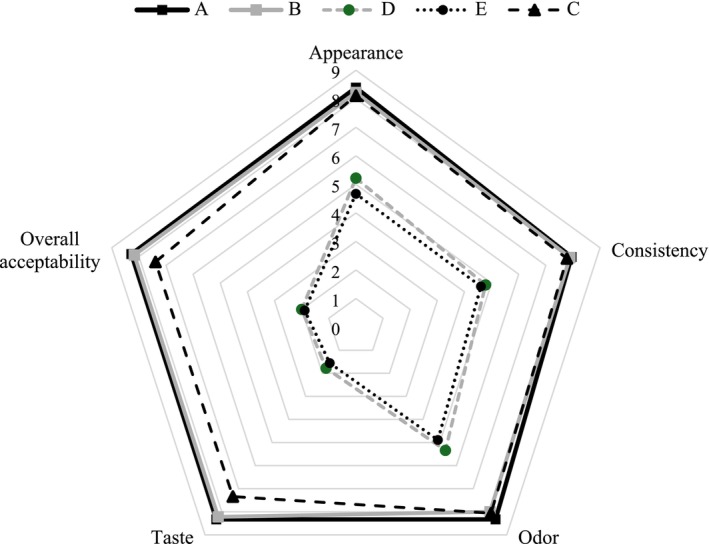
The sensory profiles of the yoghurts on the 14th day of storage. (A: Yoghurt produced by surface spraying of aqueous solutions of olive leaf extract at 0.4% concentration after incubation, B: Yoghurt produced by surface spraying of aqueous solutions of olive leaf extract at 0.6% concentration after incubation, D: Yoghurts produced by adding 0.4% powdered olive leaf extract before incubation, E: Yoghurts produced by adding 0.6% powdered olive leaf extract before incubation, C: Control yoghurt).

Figure 5 shows sensory evaluation results of all yoghurt samples on the 14th day of storage. The results indicated that yoghurts A and B had very close scores to plain yoghurt. On the other hand, all the sensory parameters of the yoghurts D and E, to which POLE were added before incubation, were dramatically lower than the others. While yoghurts A, B, and control received scores of 8.39, 8.22, and 8.11 from the panelists in appearance, respectively, these values dramatically decreased to 5.22 and 4.67 for yoghurts D and E. The color evaluation results of the present study support the appearance scores (Table 3). That is, the samples in which POLE aqueous solutions were sprayed on the yoghurt surface were very close to plain yoghurt in brightness and yellowness, while the yellowness increased, and the brightness decreased significantly in the yoghurt to which POLE was added. This can be attributed to the natural compounds found in the composition of POLE. In fact, natural olive polyphenol extracts are characterized by yellow‐brown color (Georgakouli et al. 2016). In addition, in the aqueous extract application, the concentration of olive leaf extract concentrate in the solution was quite low, which provided yoghurts A and B to give values close to the control. In the evaluation of consistency and odor, all yoghurts showed the same results as in the appearance scores.

Olive leaf extract exhibits a bitter aftertaste resulting from the natural phenolics present in its composition, mainly oleuropein, tyrosol, and hydroxy tyrosol (Tavakoli et al. 2018). This bitter aftertaste was evident in yoghurts D and E, which received the consumers' lowest scores of 1.78 and 1.56. Similarly, Barukčić et al. (2022) noted that adding olive leaf extract into yoghurt negatively affected organoleptic properties. Tavakoli et al. (2018) reported in a study that the undesirable properties in taste and color caused by free form extracts of olive leaf phenolics in yoghurt were minimized by the encapsulation process. In this context, the application of spraying POLE aqueous extract on the yoghurt surface provided similar results with the literature findings (Tavakoli et al. 2018). In both treatments, the two different concentration levels selected for POLE did not reveal significant changes in the sensory properties of yoghurt. As reflected in the evaluation scores of all sensory parameters, the yoghurts with the highest overall acceptability were A and B, and the lowest were D and E. Moreover, unlike previous studies incorporating olive leaf extract into yoghurt, this study demonstrates that surface spraying after fermentation can preserve sensory qualities while delivering functional benefits. This approach increases product quality by preserving sensory acceptability and has the potential to shed light on large‐scale industrial applications for similar natural extracts. Also, in the broader context of food preservation and sustainable food processing, our findings may contribute to the ongoing efforts to replace synthetic preservatives with safer, plant‐based alternatives.

## 4 Conclusions

This study evaluated the potential of POLE as a natural antioxidant and antimicrobial agent in yoghurt production through different application methods and concentrations. The incorporation of POLE before fermentation significantly enhanced the TPC and antioxidant activity, likely due to higher extract concentration and better integration of phenolics such as oleuropein into the yoghurt matrix. However, this method negatively affected sensory attributes. In contrast, surface spraying of POLE after fermentation improved sensory and textural properties and yielded results closer to the control, though it provided limited enhancement of functional properties. Despite this, the surface application method proved to be a novel and promising approach, effectively preventing mold and yeast growth during storage without interfering with yoghurt fermentation. These findings support the application of spraying aqueous solutions of POLE to preserve microbial quality while maintaining sensory acceptability. In this respect, it fills the existing gaps in literature and sheds light on industrial applications. Future studies should focus on optimizing extract concentration and application strategies to improve functional benefits and expand its use across various fermented dairy products.

## Author Contributions

**Büşra Deniz Aydın:** conceptualization (equal), data curation (equal), formal analysis (equal), investigation (equal), methodology (equal), software (equal), validation (equal), visualization (equal), writing – original draft (equal), writing – review and editing (equal). **Çiğdem Konak Göktepe:** investigation (equal), methodology (equal), software (equal), writing – original draft (equal), writing – review and editing (equal). **Nihat Akın:** conceptualization (equal), data curation (equal), funding acquisition (equal), project administration (equal), resources (equal), supervision (equal), validation (equal).

## Conflicts of Interest

The authors declare no conflicts of interest.

## Data Availability

The datasets generated during and/or analyzed during this study are available from the corresponding author upon reasonable request.
